# Metabolic modelling in the development of cell factories by synthetic biology

**DOI:** 10.5936/csbj.201210009

**Published:** 2012-11-12

**Authors:** Paula Jouhten

**Affiliations:** a VTT Technical Research Centre of Finland, Tietotie 2, 02044 VTT, Espoo, Finland

**Keywords:** simulation, chassis, flux, constraint-based, kinetics

## Abstract

Cell factories are commonly microbial organisms utilized for bioconversion of renewable resources to bulk or high value chemicals. Introduction of novel production pathways in chassis strains is the core of the development of cell factories by synthetic biology. Synthetic biology aims to create novel biological functions and systems not found in nature by combining biology with engineering. The workflow of the development of novel cell factories with synthetic biology is ideally linear which will be attainable with the quantitative engineering approach, high-quality predictive models, and libraries of well-characterized parts. Different types of metabolic models, mathematical representations of metabolism and its components, enzymes and metabolites, are useful in particular phases of the synthetic biology workflow. In this minireview, the role of metabolic modelling in synthetic biology will be discussed with a review of current status of compatible methods and models for the *in silico* design and quantitative evaluation of a cell factory.

## Introduction

**Figure 1:**
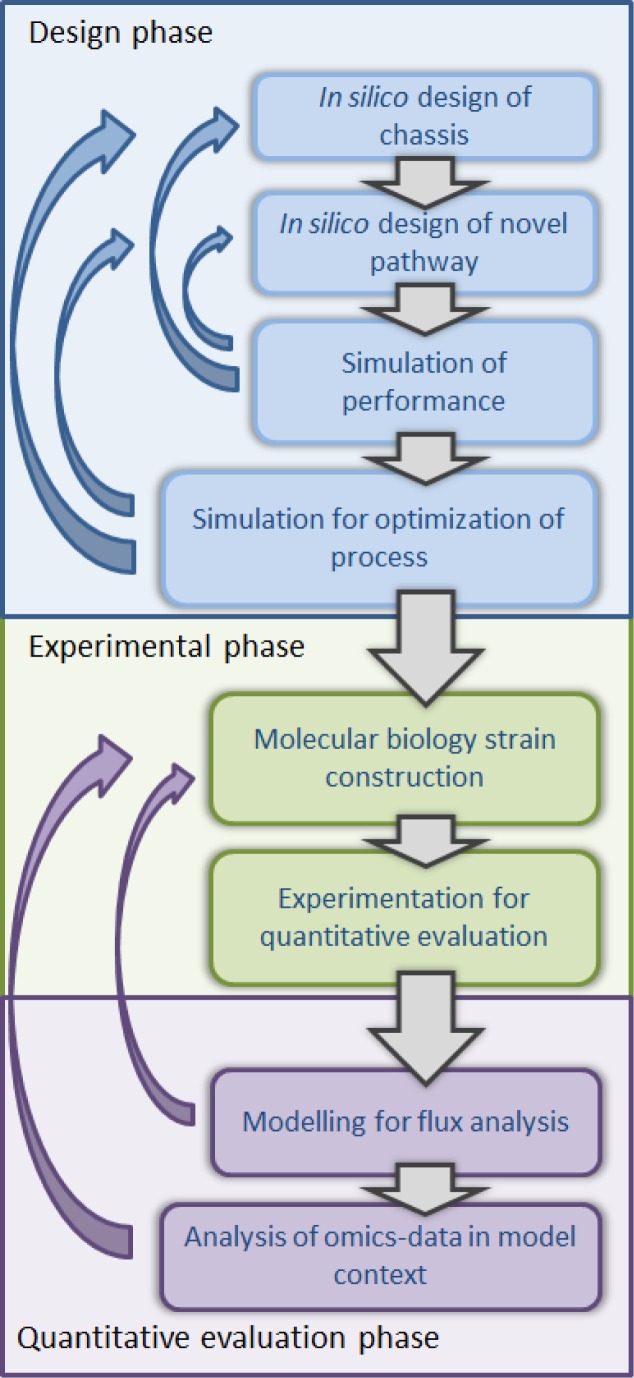
**Workflow of the development of a cell factory with synthetic biology**. The workflow of the development of a novel cell factory with synthetic biology is divided in three main phases: the design phase, the experimental phase and the phase of the quantitative evaluation of the novel strain. Metabolic modelling is involved in the design and quantitative evaluation phases. Albeit the aim for linearity in the workflow, simulated behaviour of the *in silico* strain or *in vivo* behaviour of the novel strain may force return to previous steps.

Cell factories, central to a bioeconomy, are commonly microbial organisms harnessed for bioconversion of renewable sources to bulk or high value chemicals or alternatively for carbon capture to oppose climate change. Introduction of novel, non-native pathways producing a target compound in optimized chassis strains is the core of the development of cell factories by synthetic biology. Synthetic biology aims to create novel biological functions and systems not found in nature by combining biology with engineering. Engineering provides quantitative *in silico* design and quantitative evaluation of novel biological functions and systems. The *in silico* design and the novel cell factory become described with quantitative measures in contrast to qualitative descriptions. Modelling of metabolism is involved in both design and evaluation of the novel cell factories. Modelling of metabolism, or its component metabolites and reactions, provides a simplified representation of the reality. In computational applications, a model is a quantitative, mathematical, representation of the biological systems or components on a suitable level of simplification. Different mathematical representations of metabolism, metabolic pathways, or metabolic components, enzymes and metabolites, are useful in different phases of the development of a cell factory by synthetic biology. In this minireview, the role of metabolic modelling in the emergence of synthetic biology will be discussed with a review of the current status of compatible methods and models for the design and evaluation of a cell factory. The workflow of the development of novel cell factories with synthetic biology is ideally linear with subsequent steps traversed in contrast to an iterative cycle of conventional strain improvement. The ideal, linear, workflow can be attained with the quantitative engineering approach using high-quality predictive metabolic models, and libraries of well-characterized parts. The workflow of the development of a novel cell factory can be divided in the design phase, the phase of the evaluation of a novel strain, and a strain construction and experimentation steps in between the former, Figure 1. In the design phase modelling of metabolism is required in the *in silico* design of the chassis, and the modelling of pathway components is required for the simulation of novel, optimally orthogonal, production pathways. Further, modelling of the metabolic entity consisting of the designed pathway within a chassis enables the simulation of the performance of the alternative designs in the cell (third step in the *in silico* design phase in Figure 1. Simulation can also be applied to optimize the process set up and the medium composition for the novel strain before entering the experimental strain construction phase, Figure 1. Regardless of the aim for linearity in the workflow and the availability of a high-quality predictive models in an ideal case, the simulation steps may identify properties of the *in silico* strain, which require further modifications in the design steps (blue arrows in Figure 1). When an optimal *in silico* design is ready, the strain will be constructed in the experimental phase. Experimentation will then be performed to collect data on the *in vivo* behaviour of the novel strain. After the experimental phase, metabolic modelling is needed again. Analysis of intracellular fluxes in the quantitative evaluation phase requires metabolic modelling. In addition, the quantitative omics-data collected in the experimental phase can be interpreted and the regulatory activities inferred in the context of metabolic models. If the evaluation phase reveals unsatisfactory performance of the constructed strain which considerably differs from the behaviour of the designed *in silico* strain, a return to the experimental phase is forced (purple arrows in Figure 1). The discrepancies are assumed to rise from the gap between the *in silico* design and the strain construction. Unfortunately, the *in silico* design is not yet performed on the level of the actual parts used in the strain construction by molecular biology.

**Figure 2:**
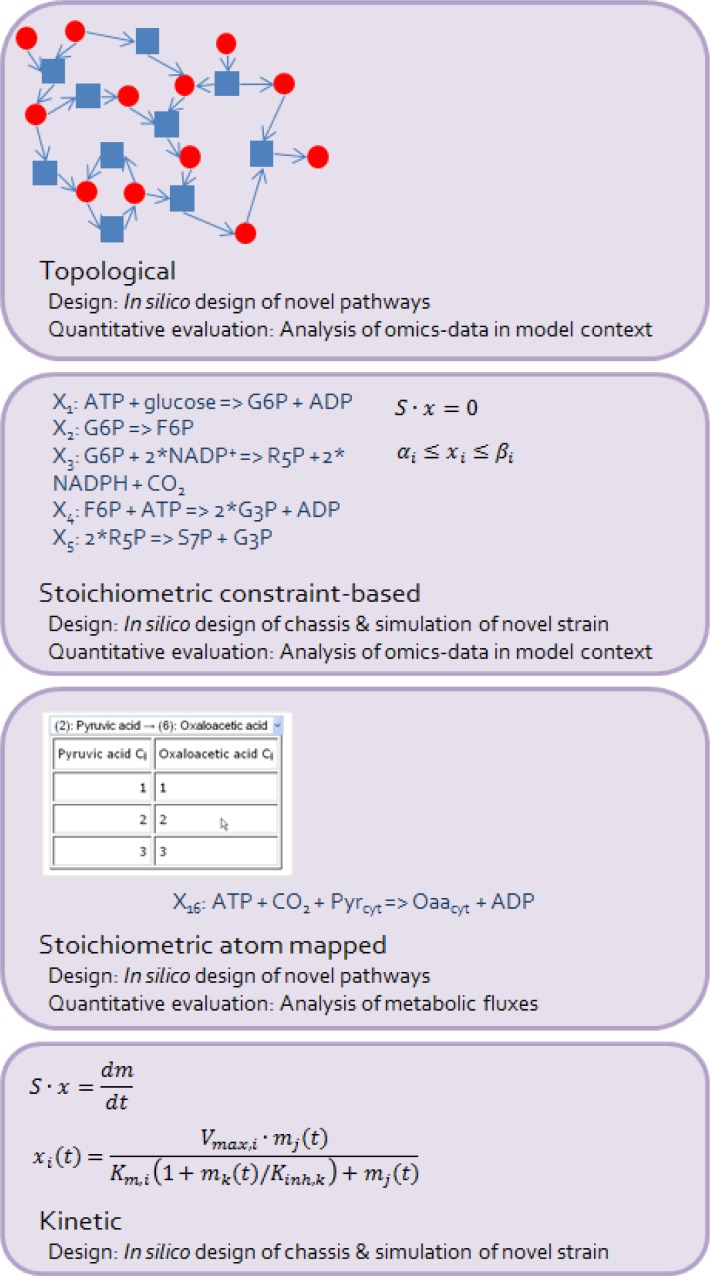
**Metabolic models involved in the development of cell factories with synthetic biology**. The four metabolic modelling approaches involved in the workflow of the development of a cell factory with synthetic biology are shown in an order of increasing complexity from topological to constraint-based, atom-mapped, and kinetic models. The phases and the tasks of the synthetic biology workflow, where the particular models are used, are shown below the descriptions of the types of the models.

Each of the steps of the synthetic biology workflow that involve metabolic modelling will be addressed in the following chapters including the promising directions, studies, and development in the field. In addition the different types of metabolic models applicable in the workflow (Figure 2) will be reviewed.

## Metabolic models involved in the development of cell factories using synthetic biology

Different types of metabolic models involved in the development of novel cell factories using synthetic biology are shown in Figure 2. The model types are presented in the order of increasing complexity. The scale of metabolism which is feasible to describe with the particular models increases in the opposite direction. Topological models include either undirected or directed interactions between the metabolites and enzymes and are being used as scaffolds for the analysis of omics-data and in path finding by graph-theoretical methods. Constraint-based stoichiometric models are static models with wide application in target identification for strain modification. These models include stoichiometric relationships between substrates and products in the reactions and reaction direction, or flux constraints. Reaction direction and flux constraints can be obtained from, for example thermodynamics and maximum enzyme capacities. Some of these models are annotated with gene-reaction rules, which provide a direct link to the genome and enable simulations of the effect of modifications of single genes. Atom-mapped stoichiometric models include mappings of atom transfers in the metabolic reactions. For applications including ^13^C flux analysis, carbon atom mappings are necessary. Kinetic models include dynamic rate equations of metabolic reactions, either as simplified or mechanistic equations. Simplified rate equations aim to reproduce the main features of enzyme reaction dynamics with limited parameters and complexity. Mechanistic rate equations describe in detail the dynamic behaviour of a reaction as it is dependent of the enzymatic mechanism.

## Design: *in silico* design of chassis

Central carbon metabolism, with its capacity for high pathway fluxes, forms the core of a cell factory. The efficient pathways of the central carbon metabolism produce the precursors for all natural chemicals [1], and this can be exploited in a chassis. Engineering a high flux to convert carbon sources to a specific precursor will provide a ready platform strain for a number of different products [2]. Usually the redirection of native pathway fluxes in central carbon metabolism involves conventional metabolic engineering, but synthetic biology approaches could provide novel solutions. Stoichiometric metabolic modelling is an established means to identify targets for metabolic engineering. Constraint-based modelling (Figure 2), using static stoichiometric models [3], is directly extendable to genome-scale which enables consideration of important genome-wide phenomena such as energy metabolism and cofactor balancing in the *in silico* design of chassis (Figure 1, Design phase). In constraint-based modelling the stoichiometric models are augmented with absolute magnitude constraints and flux direction constraints from, for example, thermodynamics [4]. The stoichiometry and the flux constraints form a feasible solution space where the true flux distribution solution lies. Flux balance analysis (FBA) uses linear programming with a defined objective function to identify an optimal flux distribution within the feasible space. Computational analysis enables also an identification of such targets for metabolic engineering which are not intuitively identifiable just by studying network maps. Extensions of FBA and other *in silico* methods based on stoichiometric models have successfully been applied to identify metabolic engineering targets in genome-wide networks. In 2009 Asadollahi *et al*. identified *in silico* *gdh1* encoding an NADPH dependent glutamate dehydrogenase as a knock-out target for enhanced synthesis of sesquiterpenes in *Saccharomyces cerevisiae* [5]. The target identification was based on stoichiometric modelling and was pursued by combining OptGene modelling framework with the minimization of metabolic adjustment (MoMA) [6] as an objective function. The *in silico* analysis predicted a ten-fold increase in the production flux, whereas the *in vivo* increase was less but still substantial. Another approach was followed by Becker *et al*. (2011) who simulated the optimal flux distribution for L-lysine production in *Corynebacterium glutamicum* and compared it to the *in vivo* flux distribution determined by ^13^C flux analysis to determine the differences for targets of strain improvement [7]. In general, the *in silico* methods for stoichiometric and constraint-based models allow for the predictions of particular optimal flux distributions, and optimizations of gene deletions, additions, and overexpression in the aim of improving yield and flux to the target product or coupling the target molecule production to growth. The different algorithms for *in silico* target identification with stoichiometric models have been thoroughly reviewed by Park *et al*. in 2009 [8]. Since then, the algorithm development has further improved the speed [9, 10] and provided methods for improved predictions (FVSEOF with grouping reaction constraints [11]; CASOP [12]; Flux Design [13]). In addition, Cobra Toolbox v.2.0 currently provides many of the existing tools in it [14]. Another software platform for the *in silico* strain engineering is OptFlux which is Matlab independent in contrast to Cobra Toolbox [15].

The purely stoichiometric metabolic modelling has weaknesses. It cannot take into account the dynamic interactions of reactions, or the metabolite concentrations and the effects of their allosteric regulation in the system. Mechanistic kinetic models (Figure 2) include all the above mentioned but are hard to parameterize in large-scale. Specific experimental data for the estimation of parameters is either time-consuming or impossible to produce and the *in vitro* data may not correctly represent the *in vivo* system behaviour. In a moderate scale the mechanistic kinetic models can be used to identify the product flux controlling reactions by the means of metabolic control analysis (MCA) [16, 17]. MCA framework can also manage the uncertainty in the system parameters [18–19]. Metabolic ensemble modelling (MEM) [20] with approximative kinetics manages the uncertainties both in the model structure and in the parameter sets. MEM has successfully been applied in metabolic engineering tasks [21]. Moreover, first attempts have been made to extend kinetic models to large-scale. Smallbone *et al*. (2010) implemented approximative linlog kinetics [22] in the reactions of uncompartmentalized, genome-scale, model of *S. cerevisiae* and were able to parameterize the model [23]. Different simplified mechanistic rate laws of enzyme reactions (for example linlog [22], convenience kinetics [24], and power laws [25]) have been developed to limit the complexity and the number of parameters required. While providing simplification, the ability to still reproduce the main features of enzyme reactions, such as saturability, is important for the approximative rate laws. The simplified rate laws have also been combined with mechanistic rate laws for part of the reactions in a model [26, 27]. Costa *et al*. (2010) gained good dynamic performance of an *Escherichia coli* model with a combination of mechanistic and linlog rate laws [26]. Linlog kinetics outperformed other combinations of simplified rate laws like generalized mass action, convenience kinetics, and power-law. Far-from-equilibrium reactions are more important for the prediction of the system behaviour to be described exactly with mechanistic rate laws than the reactions operating closer to equilibrium. Far-from-equilibrium and close-to-equilibrium reactions have been identified *in vivo* in *S. cerevisiae* by Canelas *et al*. (2011) [28].

Industrially widely utilized organisms are soft choices also as chassis organisms since their behaviour and the means for their engineering are well known. However, organisms beyond the classical producers of chemicals such as *E. coli*, *S. cerevisiae*, and *C. glutamicum*, offer important properties like high tolerance (L-valine tolerant uncommon *E. coli* engineered to a platform strain [29]; solvent tolerant *Pseudomonas putida* S12 engineered to an L-tyrosine producing platform strain [30]; *Bacillus subtilis* able to modify the cell wall in response to toxicity [31]), ability to perform carbon capture like cyanobacteria [32] and acetogens [33], or heterosynthetic properties like a possibility for electrosynthesis [34]. Genome-wide metabolic reconstructions are already available for modelling and analysis of many of these potential chassis organisms (a cyanobacterial model of *Synechocystis sp*. PCC 6803 [35]; *B. subtilis* model iBsu1103 [36]). In addition, methods for the reconstruction of metabolic models from genome data exist and they are automated to large extent [37–39].

## Design: *in silico* design of novel pathways

Computational methods offer speed and an ability to combine huge amounts of information to *in silico* design of novel pathways [40] (Figure 1, Design phase). Conventionally the metabolic path finding has been applied to search short or efficient pathways from a source metabolite to a target metabolite within the metabolic network of a single organism. However, when designing novel, heterologous, pathways the search space of reactions cannot be limited to a metabolic model of a single organism but could be extended to include all known biochemical reactions or even novel biochemical conversions. Availability of enzyme mechanisms could be considered to form an ultimate limit for the choice of novel biochemical conversions that can be engineered into cells. BNICE method developed by Hatzimanikatis *et al*. (2005) restricts the pathway search on biochemical conversions enabled by generalized enzyme reaction rules [41]. The rules are modelled, based on the EC classification system, thereby eliminating mechanistically infeasible reaction steps. Yet the generalization of the reaction rules leaves a possibility to create enhanced activities by protein engineering. Brunk *et al*. (2012) picked candidate enzymes for a novel pathway identified with BNICE and continued with structural *in silico* 3D modelling and simulation of the candidate enzymes to identify modification targets to improve the catalytic properties of the enzymes [42].

The existing methods for computational path finding divide into two general classes, steady state -based and graph theoretical frameworks. The steady state -based methods rely on optimization, or on the enumeration of elementary flux modes (EFMs) in stoichiometric models (Figure 2) [43]. Despite the recent advancements [44–46] the enumeration of elementary flux modes is computationally demanding in large networks. To design a novel pathway into a chassis the enumeration of EFMs need to be performed in a huge network containing all the endogenous reactions of an organism and all possible heterologous reactions. Carbonell *et al*. (2012) suggested introduction of additional constraints for not to waste computational power in the enumeration of EFMs having zero fluxes through the pathway leading to the target compound [47]. They also modelled the availability of a variety of source molecules into the EFM framework [47]. Other steady state -based methods aim to identify an optimal pathway design instead of the enumeration of alternative pathway designs. OptStrain method developed by Pharkya *et al*. (2004) identifies stoichiometrically advantageous deletions to the chassis in addition to the identification of optimal heterologous reactions [48]. Strength of the steady state -based methods is that they are directly able to generate branched pathway solutions and utilize a set of all endogenous metabolites whereas many of the graph theoretical methods search solely for linear paths in topological metabolic models (Figure 2). Many graph theoretical methods also require user to distinguish cofactors from other metabolites. On the other hand, the graph theoretical methods outperform the steady state -based methods in speed and they scale well into large networks. Recently, a graph theoretical method able to enumerate branched pathways from a set of source metabolites to target metabolites was developed where hyperpaths are searched in a hypergraph [47]. Already earlier a graph theoretical method for finding branched pathways was presented [49]. This method suggested by Pitkänen *et al*. (2009) successfully applied atom tracing in the path finding [49]. Atom tracing from substrate metabolites to products avoids unrealistic connections in the identified pathway and simultaneously obviates the need to define a set of cofactors or hub metabolites [49, 50]. While the topological models as such do not contain information on the reaction stoichiometry, it becomes implemented when atom tracing is utilized. Path finding methods that apply the tracing of atoms have relied on atom mappings stored in the databases of known biochemical reactions such as KEGG Rpair [51]. However, Heinonen *et al*. (2011) have recently developed an efficient computational method for atom mapping which is compatible even with novel reactions lacking database information on the atom mappings [52]. The novel method could promote the advantages of atom tracing in the *in silico* design of novel pathways.

Utilization of enumerative methods in the *in silico* pathway design tend to result in huge numbers of routes in contrast to a single design provided by the optimization based methods. The benefit is that the alternative designs could include solutions having superior properties not taken into account in the optimization. Yet it is valuable to embed some selection for feasibility into the enumeration phase, and post-ranking is required to choose the pathways to be introduced into the chassis. Ranking criteria is somewhat context-dependent but the feasibility in terms of pathway thermodynamics is generally essential for successful function in the chassis. Henry *et al*. (2010) applied thermodynamic metabolic flux analysis (TMFA) [53] to novel pathways within the stoichiometric metabolic model of the chassis [54]. TMFA utilizes a group contribution method [55] in the estimation of the free energy changes of reactions. Group contribution method estimates the free energy change of the formation of a molecule based on the structural groups it contains. Recent extension to the group contribution method by Noor *et al*. (2012) includes the consideration of the pseudoisomers of the molecule's structural groups, which gains accuracy in the estimation [56]. Feasibility of novel pathways is affected also by other factors than thermodynamics. The novel pathways can be further ranked according to the stoichiometric yields, and maximum activities of the target molecule. Both are directly obtained from TMFA and are of primary interest in the development of bioprocess strains. In the recent method developed by Chatsurachai *et al*. (2012) the yields of target molecules are evaluated at maximum growth rate [57]. Thus, the method assumes that the novel pathway is not orthogonal but dependent on the energy generation and cofactor balancing in the chassis and, therefore also on the native regulatory system. In many microbes the regulatory system is thought to manage the utilization of resources in the aim of maximizing the growth rate. However, the engineered strains may not behave like the wild type strains evolved to grow optimally and perhaps using the minimization of metabolic adjustment (MoMA) [6] as an objective function would provide more realistic predictions [5]. Further properties which affect the feasibility of engineering a pathway are, for example, the number of reactions, the number of heterologous enzymes, and the number of enzymes not directly available for expression. In general the number of heterologous enzymes counts since introduction of additional enzymes may cause a metabolic burden. In their recent review, Bar-Even *et al*. (2011) pointed out the additional importance of the characteristics of pathway intermediates such as toxicity, reactivity, and permeability [58]. In many cases the unwanted characteristics could be counteracted with spatial organisation, namely co-localization, of the pathway. To co-localize the reactions, the enzymes can be physically attached to each other with a specific linker [59]. Alternatively the reactions can be co-expressed in compartments separated from cytosol, like in peroxisomes, or in synthetic compartments, like carboxysomes [59]. Even if the metabolic model is not spatial, designed spatial organisation of the novel pathway need to be taken into account in the modelling. For example the channelling phenomenon, which may occur in a co-localized pathway, affects the distribution of flux to alternative pathways.

Lack of compatible regulatory mechanisms in the host may further hamper the production of target molecules with pathways encoded by multiple heterologous genes [60]. Design of the regulatory systems should occur in parallel with the design of the non-native pathways. If the novel pathway is composed according to the principles of orthogonality, i.e. independence of the host's native regulation, a designer made regulatory circuit could be used to optimize the function of the pathway. Designer made metabolic regulators could also be introduced to enhance viability when producing toxic compounds [61]. Orthogonal and truly constitutive promoters suitable for fine-tuned expression are desired for the cell factory applications. Diversification of natural, well-characterized, promoters could provide a source of orthogonal eukaryotic regulators [62]. In addition, orthogonal eukaryotic transcription factors have recently been designed using diversified zinc finger DNA binding domains [63].

## Design: metabolic and process simulations of the designed strain

Since microorganisms tend to strive for steady state growth, dynamic flux balance analysis (dFBA) is powerful in the simulation of the behaviour of microbial cell culture under dynamic cultivation conditions [64, 65]. DFBA assumes that the intracellular metabolism reaches pseudo-steady state while the extracellular conditions vary in a slower pace. Uptake fluxes are commonly described with dynamic equations and the derivatives of extracellular product concentrations are obtained from the metabolite balancing of a stoichiometric model. Furthermore, dFBA is applicable in genome-scale as was shown in the recent publications by Vargas *et al*. (2011), Ghosh *et al*. (2011), and Jouhten *et al*. (2012) [66–68]. Therefore dFBA is also suitable for the simulation of the designed strain in the *in silico* design phase of the workflow in Figure 1. Whole system wide cofactor and energy requirements become simultaneously considered enabling optimization of the culture setup and the growth medium (Figure 1, fourth step of the *in silico* design phase). Different dynamic set ups from batches to fed-batches with variable feed programs can be simulated and optimized for high productivity or titre [69]. If, during the cultivation process, drastic changes occur in the extracellular conditions, which are known to trigger extensive remodelling of the hierarchical regulation of the chassis’ metabolism, this regulation must be included in the model. For example, changes in the concentration of glucose and/or uptake trigger reorganization of the metabolism of *S. cerevisiae*, which was recently modelled by Moisset *et al*. (2012) [70]. They introduced glucose-dependent hierarchical regulation into a dynamic model of *S. cerevisiae*. A glucose batch culture with a diauxic shift from glucose utilization to ethanol oxidation was successfully simulated with the model. Although the signalling and transcriptional regulation in *S. cerevisiae* in response to glucose is well known, the regulation of the metabolic fluxes including all the post-transcriptional and metabolic regulation has not been completely solved. Metabolic regulation also by a flux sensor which mediates the regulation of the distribution of flux to fermentative and respirative pathways in response to the flux in the upper glycolytic pathway has been suggested by Kotte *et al*. (2010) [71].

DFBA offers also means to model and simulate a consolidated bioprocess where the breakdown of polymeric resource into metabolizable molecules and the fermentation occur in a single process. The breakdown of raw material, the uptake of the metabolizable carbon source, the biosynthesis of the product by the novel strain, and the process set up can be included into a single model which enables *in silico* evaluation of different designs in complete process simulations. DFBA has been applied to a consolidated bioprocess by Salimi *et al*. (2010) who simulated the consolidated process of a co-culture of *Clostridia* species with native cellulolytic activity [72]. An example of fine strain engineering for consolidated bioprocesses was the recently performed study by Bokinsky *et al*. (2011) [73]. They engineered *E. coli* strains to secrete cellulase, xylanase, beta-glucosidase, and xylobiosidase enzymes for the breakdown of raw material outside the cells and to produce biofuel components and precursors via heterologous metabolic pathways within the cells. In a similar case the optimal compatibility of the strain modifications and the process conditions could be computationally designed by dFBA.

## Quantitative evaluation: metabolic flux analysis

After the novel strain has been constructed by molecular biology the *in vivo* performance of the strain need to be quantitatively evaluated to validate if the *in silico* designed and simulated performance has been reached (Figure 1, quantitative evaluation phase). Evaluation of the performance of a cell factory in terms of extracellular fluxes can be made by a direct measurement of an accumulation of extracellular compounds in the medium. On the contrary, the intracellular fluxes cannot be directly measured. Isotopic tracer experiments together with metabolic modelling are required to obtain information on the *in vivo* pathway fluxes (Figure 1, metabolic flux analysis –step). A stoichiometric metabolic model need to be augmented with at least carbon atom mappings of substrate atoms to product atoms for the reactions to enable *in vivo* flux estimation via isotopic tracer experiments (Figure 2). Atom mappings for known reactions are stored in a few databases (ARM database (www.metabolome.jp) [74]; KEGG ligand and rpair database [52]), and alternatively the mappings can be determined by solving the “atom mapping problem” for which Heinonen *et al*. (2011) have provided a novel computational solution [53]. For *E. coli* a fully atom mapped (all except hydrogen atoms mapped) genome-scale metabolic model for flux analysis by the global iterative fitting approach was published by Ravikirthi *et al*. (2011) [75]. Stable isotopic ^13^C labelling is commonly applied for a quantitative analysis of *in vivo* fluxes. ^13^C labelled substrate is feed to the cells after which the cells are harvested for the detection of ^13^C labelling patterns in either the metabolic intermediates or in the biomass components. The resolution of the quantitative information on the *in vivo* fluxes is strongly dependent on the choice of the ^13^C labelling strategy. The substrate molecules may be fractionally uniformly labelled, labelled in particular carbon positions of the molecule, or the labelling strategy may combine both of the previous approaches. An optimal labelling strategy for resolving a particular flux or fluxes is dependent on the structure of the metabolic network and can be addressed computationally [76]. In the recent method development Schellenberger *et al*. (2012) applied Monte-Carlo sampling of feasible flux spaces in constraint-based models [77], and Crown and Antoniewicz (2012) formulated a solution to the problem in the elementary metabolite unit's (EMU's) framework [78, 79] to select an optimal labelling strategy. The EMU formulation is the most recent and computationally efficient formulation of ^13^C labelling patterns.

^13^C labelling patterns are detected either by nuclear magnetic resonance (NMR) spectroscopy or by mass spectrometry (MS). Even though the recent hardware development of NMR spectroscopy in terms of the minimisation of thermal noise in the electronics has substantially increased the sensitivity, MS is intrinsically more sensitive method than NMR spectroscopy. The detection method options differ also in NMR spectroscopy being capable of providing direct positional information on the ^13^C label whereas MS can directly detect mass differences between molecules or molecule fragments. Fragmentation in the MS measurement enables generation of partially positional information. Identification of fragments in MS spectra is alleviated not only by database information but also by computational methods suitable for novel fragments lacking the database information (non-commercial methods [80, 81]). Fragment identification involves modelling of molecule and fragment structures and favourability of bond cleavages dependent on the structure. After the ^13^C labelling data on either the metabolic intermediates or biomass components has been acquired, the analysis of fluxes may proceed in two alternative ways: via direct local determination of relative *in vivo* fluxes through alternative pathways [82] or via global iterative fitting [83]. The relative *in vivo* fluxes through alternative pathways can also be introduced as additional constraints into a metabolic flux analysis problem to render the absolute fluxes solvable [84, 85]. Because of the local nature of the relative *in vivo* flux constraints, the obtained solution of the metabolic flux analysis problem is less prone to inaccuracies in the model structure and few ^13^C data [86, 87]. In contrast, global iterative fitting is a non-linear optimization problem where candidate *in vivo* flux distributions are iteratively generated and the corresponding ^13^C labelling patterns simulated and compared to the experimental data until the fit is satisfactory. This approach has been applied to different cell types from bacteria even to mammalian cells [7, 88–90]. There are few specific software available for the global iterative fitting approach (13C-FLUX [83]; 13C-FLUX2: www.13cflux.net; OpenFLUX [91]) which apply different formulations of the ^13^C labelling patterns. Drawbacks of the global iterative fitting approach are that the size of a metabolic system which is feasible to analyse is limited and that it is difficult to ensure that the resulting flux distribution solution is globally optimal and unique [86]. However, currently the extent of which ^13^C labelling data covers the genome-scale metabolic systems is not broad. ^13^C labelling data acquisition usually covers tens of compounds which is only a limited fraction of all the metabolites present in cells. Without this limitation the approach of using local relative fluxes as additional constraints in a metabolic flux analysis system would be extendable in genome-scale whereas global iterative fitting would become computationally heavy. Blank *et al*. (2005) approached the extension of the metabolic flux analysis into genome-scale by constraining the fluxes in a genome scale stoichiometric metabolic model with the flux solution from a smaller, central carbon metabolism scale problem [92]. More recently, Suthers *et al*. (2010) were able to decrease the computational burden of ^13^C constrained flux analysis with the implementation of the elementary metabolite unit (EMU) formulation and flux coupling into a global iterative fitting approach [93].

## Quantitative evaluation: analysis of hierarchical expression data in context of a metabolic model

Targeted quantitative analysis of protein expression has empirically observed to provide important information for balancing the reactions of a heterologous pathway [94]. In addition to the targeted analyses of expression, systems biology analyses in large-scale are of value for understanding the active mechanisms underlying the metabolic performance of a cell factory. At present only transcriptomics data can be generated truly in genome-scale. Proteomics and metabolomics are progressing but the diversity and the low amounts of the components hinder the analyses. Omics-data can be integrated into the context of a metabolic network model to infer the *in vivo* regulation (Figure 1, analysis of hierarchical expression data in model context). Integration of transcription data into a constraint-based model (Figure 2) context enables for example an identification of reactions whose transcriptional status and flux status are inconsistent which leads to an identification of the reactions being regulated at post-transcriptional level [95]. Further, the Reporter metabolite -algorithm integrates transcription data into a topological metabolic model (Figure 2) context and identifies “hot-spot” metabolites around which significant transcriptional regulation has taken place in the network [96]. These reporter metabolites aid to decipher the transcriptional regulation of metabolism and to generate hypotheses on the participation of the Reporter metabolites in the systemic regulation under the conditions studied.

## Summary and outlook

Metabolic modelling plays an essential role in the development of cell factory by the means of emerging synthetic biology. Modelling and simulation provide quantitative data on the performance of alternative designs. Especially large-scale modelling of metabolism is central when optimal designs of cell factory systems are sought. In particular the constraint-based modelling scales well in genome-scale whereas dynamic models in large-scale are only emerging. Thus, the constraint-based models have successfully been applied in the identification of engineering targets. On the other hand, the shortcomings are also being confessed. In constraint-based models the detailed dynamic features and the regulatory dependencies on the metabolome are not implemented. However, dynamic models require a lot of experimental data for parameterisation, which is both laborious and expensive or even impossible to generate. Therefore, the modelling approaches are developing to cope with the scarcity of *in vivo* data from biological systems from which examples are modelling under uncertainty by Mišković and Hatzimanikatis (2011) [18] and similar, metabolic ensemble modelling [97]. Simultaneously simplified kinetics for reaction rate expressions are increasingly being tested and analysed for suitability for even large-scale applications [23]. Both the constraint-based and the dynamic metabolic modelling methods can be utilized to design the chassis and to simulate the performance of heterologous pathways in the chassis. Moreover, dynamic flux balance analysis, in genome-scale [66–68], is readily extendable for quantitative optimization of process conditions together with the engineered cell factory, and even for simulation of consolidated bioprocesses.

Many previously developed path finding methods are applicable to the design of novel, heterologous, production pathways and specific methods for synthetic biology are also already available. BNICE, which is capable of finding truly novel pathways, constrains the space of possible biochemical conversions only with general enzymatic reaction rules, the particular enzyme need not to be known or characterized [41]. Catalysts can then be designed and produced accordingly by protein engineering.

The need to accumulate knowledge and to improve the models by systems biology studies still remains. The ideal synthetic biology workflow, Figure 1, is reality only when the models are good enough to provide quantitatively reliable predictions. However, many promising chassis organisms are not yet known in detail. In addition, the libraries of well-characterized parts are still scarce in particular for other organisms than *E. coli*. A true establishment of synthetic biology will require supporting efforts to develop the models and parts. The iterative cycle of information generation and model improvement, which systems biology focuses on, could roll faster to support the emerging synthetic biology. Knowledge on the regulation of the metabolism should be integrated into the metabolic models to reach a good level of predictability under various conditions the cells may be exposed to. Urgency to rationalize the engineering of the regulatory systems of cell factories was recently stressed also by Yadav *et al*. (2012) [60]. Modelling is definitely a centrepiece of rationalization. What is the perfect modelling framework for the integrative models, we haven't seen yet. Adequate knowledge integrated into the models and the availability of experimental techniques and parts are prerequisites for a fast streamlined course from the design of a chassis to an optimal cell factory.
